# Advances in 3D Printed Scaffolds for Periodontal Regeneration

**DOI:** 10.1007/s40496-025-00421-7

**Published:** 2026-01-27

**Authors:** Arwa Daghrery, Igor Paulino Mendes Soares, Alexandre H. dos Reis‑Prado, Isaac J. de Souza Araújo, Renan Dal-Fabbro, Marco C. Bottino

**Affiliations:** 1 Department of Restorative Dental Sciences, School of Dentistry, Jazan University, Jazan, Kingdom of Saudi Arabia; 2 Department of Dental Materials and Prosthodontics, School of Dentistry, São Paulo State University (UNESP), Araraquara, Brazil; 3 Department of Restorative Dentistry, School of Dentistry, Federal University of Minas Gerais (UFMG), Belo Horizonte, Brazil; 4 College of Dentistry, University of Saskatchewan, Saskatoon, SK Canada; 5 Department of Cariology, Restorative Sciences and Endodontics, University of Michigan School of Dentistry, 1011 N. University (Room 2303), Ann Arbor, MI MI - 48109 USA; 6 Department of Biomedical Engineering, College of Engineering, University of Michigan, Ann Arbor, MI USA

**Keywords:** Periodontal regeneration, Periodontitis, 3D printing, Bioprinting, Biomaterials, Tissue engineering

## Abstract

**Purpose of Review:**

To compile recent advances in scaffold-guided periodontal regeneration (SGPR) enabled by 3D printing, focusing on innovations in materials, multiphasic/anisotropic design, image-guided personalization, and the spatiotemporal delivery of therapeutic cues.

**Recent Findings:**

Composite scaffold systems, extracellular matrix (ECM)-mimetic hydrogels, and ion-releasing ceramics (e.g., Mg/Sr/Ca-phosphates, bioactive glass, among others) enhance osteogenesis, periodontal ligament (PDL) formation, and angiogenesis. Melt electrowriting, extrusion, and inkjet printing enable the creation of patient-specific, multiphasic and anisotropic scaffolds that mimic cementum-PDL-bone interfaces. Controlled release of ions, growth factors, genes, and antimicrobials modulate immunity and microenvironments. Emerging directions include in situ and 4D bioprinting, immuno-instructive and prevascularized constructs, and CAD models derived from clinical imaging, which are essential for manufacturing personalized scaffolds and grafts.

**Summary:**

3D printing is advancing SGPR toward functional, personalized therapies; however, its translation depends on reliable vascularization, immune modulation, long-term mechanics, scalable manufacturing, and clear regulatory and safety pathways. Standardized workflows, hybrid/4D printing, machine-learning-guided design, and rigorous clinical studies are essential to accelerate clinical adoption.

## Introduction

Periodontal disease is a common, chronic inflammatory condition characterized by the progressive destruction of the supporting tissues of the teeth, including the gingiva, periodontal ligament (PDL), cementum, and alveolar bone, which can ultimately result in tooth loss if not treated [1]. Its complex cause, involving both harmful biofilms and a dysregulated immune response, presents significant treatment challenges. Conventional periodontal treatments, such as scaling, root planing, and flap surgeries, aim to control the disease but do not fully regenerate the lost tissues [1, 2].

Unlike craniofacial or musculoskeletal tissue regeneration, which often focuses on restoring a single mineralized tissue with relatively uniform architecture and load-bearing requirements, periodontal regeneration requires the simultaneous and spatially organized reconstruction of multiple, compositionally and functionally distinct tissues, including alveolar bone, cementum, and the PDL. Importantly, the dominant regenerative challenges differ between the alveolar bone and the PDL. Alveolar bone regeneration primarily involves rebuilding a mineralized, vascularized tissue capable of restoring defect volume and providing mechanical support, which depends on adequate osteoconduction/osteogenesis, angiogenesis, and controlled mineral deposition within a confined defect space. In contrast, PDL regeneration requires re-establishing a thin, highly specialized non-mineralized fibrous connective tissue with oriented collagen fiber bundles that insert into both newly formed cementum and alveolar bone, preserving the PDL space and enabling physiological load dissipation. Because functional PDL attachment is defined not only by tissue presence but by fiber organization and insertion, incomplete or misdirected regeneration can result in ankylosis, root resorption, or formation of a long junctional epithelium rather than true functional attachment [1, 2].

In recent years, scaffold-guided periodontal regeneration (SGPR) has become an increasingly popular approach to overcoming these limitations. Scaffolds designed for SGPR aim to mimic the natural extracellular matrix (ECM) by offering a supportive three-dimensional (3D) framework that encourages cellular adhesion, proliferation, differentiation, and blood vessel growth [3, 4]. However, unlike scaffolds used for calvarial or long-bone defects, periodontal scaffolds must operate within a confined, bacteria-rich oral environment, withstand cyclic occlusal forces, and integrate seamlessly across soft-hard interfaces. In practice, this means scaffold designs must often address bone-facing requirements (space maintenance, osteogenic cues, mineralizable matrices) while simultaneously enabling PDL-facing requirements (microscale guidance cues and anisotropic architectures to direct fiber alignment and prevent mineralized bridging). Unlike traditional passive barrier membranes used to prevent epithelial downgrowth, modern scaffolds are engineered to actively influence tissue regeneration through their mechanical properties, biochemical signals, and spatial configuration, tailored to the anatomy of the periodontal defect [5].

**Fig. 1:**
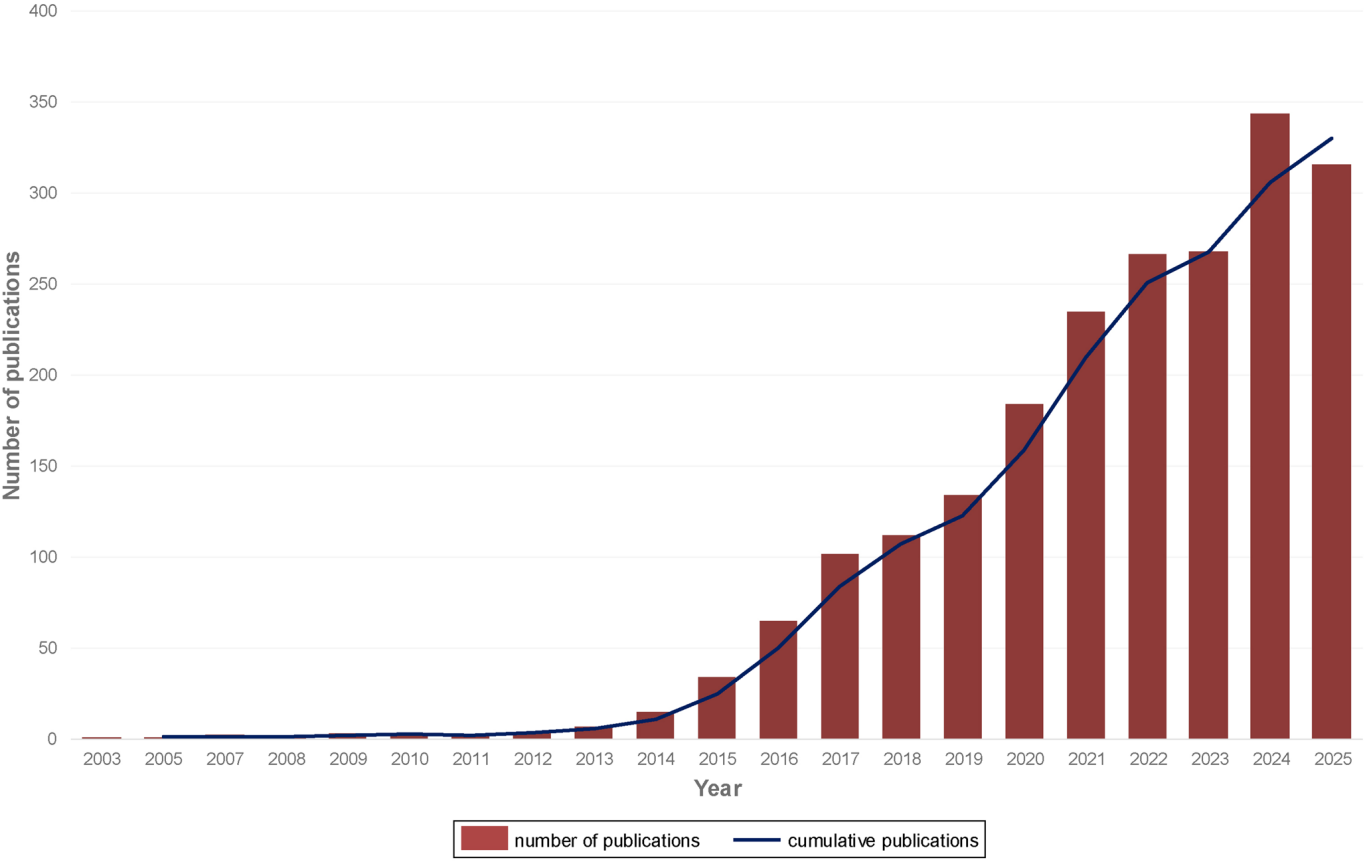
Growth of publications on 3D printing for periodontal regeneration (2003–2025). Annual publication counts (bars) and cumulative total (line) derived from a PubMed search conducted in September 2025 using: (“periodontal regeneration” OR “periodontal tissue regeneration” OR “guided periodontal tissue regeneration” OR “periodontal guided tissue regeneration” OR “periodontal ligament regeneration” OR “guided tissue regeneration” OR “bone regeneration”) AND (bioprinting OR “biofabrication” OR “three-dimensional printing” OR “printing, three dimensional” OR “3D bioprinting” OR “3D Printing” OR “3D Printings” OR “3-dimensional printing” OR “3D-printed scaffolds” OR “3D bioprinting technology” OR “3D-printed bone graft” OR “3D-printed membrane” OR “melt electrowriting” OR “extrusion-based 3d bioprinting” OR “extrusion-based printing” OR “inkjet bioprinting” OR “inkjet-based bioprinting” OR “inkjet-based printing”). Total records retrieved: 1,863

The integration of additive manufacturing (AM) with regenerative strategies has further revolutionized scaffold fabrication. 3D printing enables precise customization of scaffold features, such as pore design, mechanical properties, and controlled release of bioactive agents, enabling the creation of patient-specific constructs with high anatomical accuracy [4]. Nevertheless, translating AM technologies from musculoskeletal applications to periodontal regeneration remains challenging, as periodontal scaffolds must achieve microscale resolution to guide PDL fiber alignment, incorporate multiphasic interfaces, and degrade in a coordinated manner that matches the healing kinetics of each periodontal tissue. More advanced applications, such as bioprinting of live cells and multi-tissue interfaces, aim to replicate the hierarchical structure of periodontal tissues, particularly the soft PDL between mineralized cementum and bone interfaces [3, 5, 6]. Reflecting this progress, a PubMed survey conducted in September 2025 returned 1,863 records on 3D printing for periodontal regeneration, with annual publications steadily increasing since 2003 (Fig. 1).

This review maps current advances in SGPR, with a focus on materials innovation and biofabrication. It covers composite, hybrid, and bio-inspired biomaterials, such as PCL blends, ECM-mimetic hydrogels, ion-releasing ceramics, and smart systems that integrate printability, stability, degradation, and immunomodulation at the cementum-PDL-bone interface. We analyze advanced 3D printing techniques (extrusion, inkjet, melt electrowriting, light-based lithography) and design principles for multiphasic, anisotropic, and patient-specific constructs, and we discuss manufacturing, regulatory/safety, and clinical adoption barriers that must be addressed for translation.

## Materials Innovation for Scaffold Development

The applicability and functionality of periodontal tissue regeneration strategies rely on the basic principles of the tissue engineering triad (scaffolds, biomolecules, and cells). Guided Tissue Regeneration (GTR) and Guided Bone Regeneration (GBR) procedures have been performed to treat periodontal defects since the late 1980s, in combination with conventional root scaling and planning [5, 7–10]. GTR and GBR rely on barrier membranes to create space for selective repopulation of stem cells to regenerate lost periodontal structures. However, their regenerative outcomes remain limited, with measurable yet insufficient improvements that do not fully restore function [1, 4].

Over the years, many biomaterials have been used in periodontal regeneration, progressing from non-resorbable options (Polytetrafluoroethylene (PTFE), titanium-reinforced PTFE) to synthetic (polycaprolactone, polylactic acid (PLCL)) and naturally-based (collagen, gelatin, hyaluronic acid) resorbable compounds [9, 10, 13]. The properties and nature of these materials influence tissue response and therapeutic outcomes, and specific criteria must be met for their use in periodontal regeneration and implant dentistry [14]. Additionally, the complex architecture of periodontal tissues, which have hard-to-soft transitions that regenerate at different rates, highlights the importance of using materials with characteristics (such as printability, mechanical stability, biodegradability rate, biocompatibility, and bioactivity) compatible with available technologies. This section discusses recent findings and current ideas related to advanced materials for SGPR.

### Bioceramic Materials

The clinical success of SGPR in treating large defects, preserving alveolar bone, and even performing sinus lift procedures is typically attributed to the membrane’s stability, combined with inorganic graft materials that stimulate bone formation. Over the years, autologous bone grafts have demonstrated the greatest regenerative potential, but donor-site morbidity remains a limitation. Therefore, inorganic materials, such as hydroxyapatite, β-tricalcium phosphate, magnesium phosphate, and various bioglasses, have been systematically evaluated for the treatment of periodontal defects. The use of 3D-printed and personalized bioceramic materials for craniofacial regeneration has recently been reviewed elsewhere [11]; here, we present specific SGPR strategies using bioceramic materials.

Calcium phosphate-based materials have been among the most commonly used inorganic compounds for developing SGPR strategies. Personalized calcium phosphate-based scaffolds have been 3D-printed from CBCT scans to reconstruct alveolar ridge defects, demonstrating good printability and accuracy for patient-specific therapies [12]. Moreover, 3D-printed TCP/HA scaffolds were implanted in vivo to augment vertical bone in mandibular defects, with successful new bone formation [13]. Besides, fluorinated calcium phosphates have previously been used as coatings to enhance osteogenesis in SGPR strategies, with in vitro and in vivo validation [14, 15]. Other sources of ions that induce osteogenesis or mineralized tissue regeneration are also tested for SGPR with promising results. For instance, scaffolds containing magnesium phosphate have been prepared using various biofabrication technologies for bone regeneration in the craniofacial area, with successful outcomes [16], including specific transition zones for the periodontal compartments [17]. Recently, bioactive glass 58 S was used to produce melt electrowritten scaffolds for bone regeneration. The scaffolds exhibited a porous structure, biocompatibility, stimulation of mineral deposition, and angiogenic potential, all of which are relevant characteristics for SGPR [18]. Bioactive glasses have also demonstrated the ability to enhance the biocompatibility and osteogenic potential of calcium phosphate-based materials, and 3D-printed bioactive glass/calcium phosphate scaffolds have been tested as bioinks for bone regeneration [19].

Apart from that, hydroxyapatite, as the major inorganic component of mineralized tissues, has been used to synthesize scaffolds for bone regeneration. Critical-sized mandibular defects were successfully healed with demonstrated new bone formation and expression of important markers of regeneration through 3D-printed hydroxyapatite-based scaffolds [20]. Several other models have incorporated hydroxyapatite into polymeric scaffolds to optimize osteogenic capacity and promote bone regeneration, as further discussed in this Sects [21–23]. Inorganic compounds, such as bioceramic materials, significantly impact tissue response in periodontal regeneration, and SGPR strategies incorporating these compounds are promising. Nevertheless, some additional considerations are important regarding the printability and handling of these materials for implantation, since they are overall brittle, and the degradability ratios must be timed to ensure proper tissue growth. Moreover, bulky scaffolds based on bioceramic materials may compromise revascularization and long-term stability of the new tissue, and combinations with other materials are a valid approach to designing reliable scaffolds that incorporate these inorganic compounds.

### Composite and Hybrid Materials

Materials designed for periodontal tissue engineering should prevent or control infection at the defect site, restrict unwanted soft tissue infiltration, and recreate native-like conditions that support coordinated regeneration, while mimicking the periodontium’s complexity [24, 25]. However, it is unlikely that a single material will present all these characteristics. In this sense, considerable effort has been devoted to developing composite and hybrid materials that can approximate most of the desired aspects for successful regeneration.

Periodontal membranes based on synthetic polymers, natural compounds, or composite blends have been designed and functionalized with agents for antimicrobial activity and mineralized tissue formation, from conventional solution-based electrospinning to high-resolution and more sophisticated 3D printing strategies, as extensively reviewed elsewhere [6, 25, 26]. Overall, polycaprolactone (PCL) is the synthetic polymer of choice for SGPR applications due to its versatility and FDA approval. For instance, PCL-based electrospun membranes were functionalized with zinc oxide to achieve dual activity (antimicrobial and osteoconductive), yielding positive outcomes in vitro and in vivo [27]. Meanwhile, functionally-graded membranes demonstrate stability and integration between layers by blending synthetic (poly(L-lactide-co-ε-caprolactone(PLCL)/ polylactic acid (PLA)) and natural (gelatin) polymers as a base for loading metronidazole for antibacterial activity and nano-hydroxyapatite for bone regeneration [28]. Other biodegradable polymers, such as poly(lactic acid) (PLA), poly-L-lactic acid (PLLA) [29], polydioxanone (PDS) [30], and poly(lactic-co-glycolic acid) (PLGA) have also been tested and functionalized for periodontal regeneration.

**Fig. 2:**
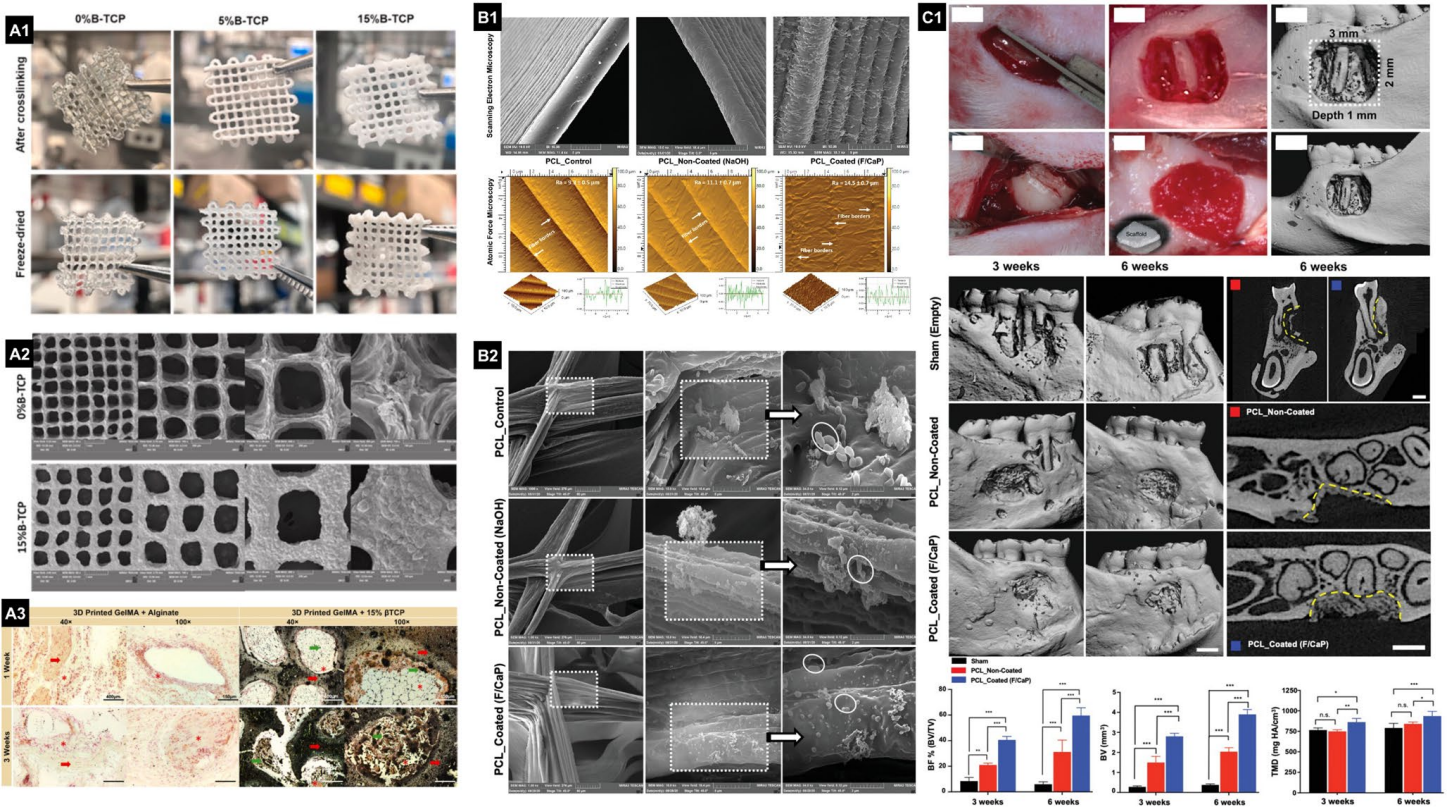
(**A1**) Digital images of 3D-printed hydrogels after crosslinking and lyophilization show slight shrinkage. (**A2**) SEM images (1000×−100×) reveal well-defined pores and the incorporation of β-TCP. (**A3**) Von Kossa staining of rat subcutaneous implants at 1 and 3 weeks shows calcium carbonate deposits, especially in the GelMA + 15% β-TCP group, where dark granular deposits within constructs indicate promoted mineralization. Printed constructs, tissue, and deposits are marked by red arrows, asterisks, and green arrows, respectively. (**B1**) SEM and AFM images show distinct surface textures of control, NaOH-etched, and F/CaP-coated MEW scaffolds, with F/CaP coatings increasing roughness. (**B2**) *P. gingivalis* growth and 2-day biofilm formation were significantly reduced on F/CaP-coated scaffolds compared to pristine and non-coated groups (*n* = 6, *p* < 0.05). (**C1**) A rat mandibular fenestration defect model (3 × 2 × 1 mm) was used to evaluate bone regeneration. The F/CaP-coated scaffold absorbed blood for clot retention and promoted superior bone volume, fill, and mineral density at 3- and 6-weeks post-implantation versus controls, confirmed by micro-CT (*n* = 4, *p* < 0.05)

From a manufacturing standpoint, thermoplastic polymers, especially PCL, offer excellent printability for complex architectures and can be readily functionalized with inorganic phases (β-TCP, hydroxyapatite, magnesium phosphate, and bioactive glasses) either as composites or surface coatings [5, 11, 14, 17, 18, 22]. Personalized PCL/hydroxyapatite scaffolds comprised of multiphases; Phase A (cementum/dentin, 100 μm), Phase B (PDL, 600 μm), and Phase C (bone, 300 μm), the scaffold promoted the formation of PDL-like fibers integrated with bone- and cementum/dentin-like tissues in subcutaneous model. Indicating that a single stem/progenitor cell population can differentiate into putative dentin/cementum, PDL, and alveolar bone complex, guided by the scaffold’s biophysical properties [22]. Exposing hydroxyapatite within PCL via alkaline erosion further enhanced osteoconduction in vivo [21]. Similarly, coating Melt electrowriting (MEW) PCL scaffolds with fluorinated calcium phosphate promoted periodontal regeneration in fenestration defects [14] (Fig. 2). Meanwhile, bioglass incorporation into PCL increased osteoinductivity while maintaining in vivo biocompatibility [18]. A practical limitation, however, is the high processing temperature of these polymers, which constrains the direct, single-stage printing of temperature-sensitive proteins or living cells.

From a therapeutic and cell-compatibility perspective, natural polymers are widely used in SGPR. For instance, collagen provides a structural framework and biochemical cues that support cell adhesion, proliferation, and tissue regeneration [31, 32]. For example, a collagen/elastin-like peptide bioink co-loaded with bioglass, doxycycline, and BMP-2 promoted mineralized tissue formation in vitro and integrated with native bone in vivo [31]. Other naturally derived matrices such as gelatin, alginate, hyaluronic acid, chitosan, and silk are broadly suitable for regenerative applications, a trend accelerated by 3D bioprinting [33]. Thermosensitive chitosan/β-glycerophosphate hydrogels functionalized with BMP-7 and ornidazole have also shown promising results in promoting periodontal tissue regeneration in class III furcation defects [34].

Naturally derived materials create ECM-mimetic microenvironments that support cell attachment and proliferation for cell-based therapies, while enabling localized and controlled delivery of bioactive molecules. In that sense, a composite bioink made of ECM and amorphous magnesium phosphate (AMP) demonstrated good potential for bone formation in vivo [16]. Additionally, silk fibroin/collagen/hydroxyapatite bioinks functionalized with erythropoietin have also been shown to produce significant alveolar bone regeneration in vivo [23]. Moreover, incorporating β-TCP into GelMA/alginate increased the compressive modulus, slowed biodegradation, and enhanced osteogenic potential without compromising in vivo biocompatibility (Fig. 2) [35]. GelMA-based bioink has also been loaded with AMP and further reinforced with MEW meshes to create mechanically stable scaffolds for load-bearing areas, while also inducing bone regeneration in vivo [36].

Functionalizing hybrid and composite systems enables precise tuning of scaffold composition and properties. However, material compatibility with 3D printing techniques varies, often necessitating multi-technology platforms or combined biofabrication strategies to achieve optimal therapeutic outcomes.

### Bio-Inspired Materials

It has been highlighted so far that SGPR should provide biological and mechanical cues for the hierarchical organization of the periodontium and its interfaces. In this regard, the formulation of bio-inspired scaffolds requires the use of materials that mimic natural conditions and/or the architecture of the periodontal microenvironment.

Compartmentalized, multiphasic scaffolds that mimic the microenvironments of cementum, PDL, and alveolar bone are increasingly used in SGPR. Functionalizing each compartment with targeted bioactive cues shows promising results in periodontal regeneration. For instance, multiphasic/tri-layered scaffolds made of chitin-PLGA-nanobioactive glass ceramic (nBGC) and functionalized with growth factors, including cementum protein 1 (for the cementum layer), fibroblast growth factor 2 (for the PDL layer), and platelet-rich plasma-derived growth factor (for the bone layer), promoted guided periodontal regeneration in rabbit maxillary defects [37].

Despite efforts in scaffold design and functionalization, regenerating the PDL with an appropriate fiber guide and orientation remains a challenging task. In that regard, micropatterned and 3D-printed micro-grooved PCL scaffolds have been fabricated to guide PDL cells toward specific alignments [38–40]. Moreover, micropatterned PCL/PLGA scaffolds treated with platelet-derived growth factor-BB and BMP-7 provided a favorable microtopography for cell alignment [41]. More recently, collagen-based bioinks loaded with periodontal ligament stem cells (PDLSCs) have been printed directly onto root fragments to rebuild the PDL compartment, resulting in favorable osteogenic activity and maintaining a PDL-compatible thickness (~ 300 μm) after 10 weeks of ectopic in vivo implantation [42].

Importantly, bio-inspired multiphasic scaffolds made from diverse materials have been engineered to deliver cells into specific periodontal compartments. Biphasic scaffolds made of PCL/β-TCP for the bone compartment and pure PCL electrospun mats for the PDL compartments were fabricated, providing biological cues to guide cellular differentiation and cementum deposition [43]. The same group later combined MEW with conventional electrospinning to fabricate biphasic scaffolds that, in a large-animal model, supported mineralized tissue formation and aligned PDL fiber development [44]. Moreover, fluorinated calcium phosphate MEW scaffolds infused with collagen were designed to replicate the organic/inorganic components of alveolar bone, favoring the stem cell response in vitro and improving bone healing in vivo [45].

Beyond the context of healthy tissues, systemic diseases, particularly diabetes, are well known to impair healing and lower the success of periodontal therapy. To promote the normal bone-healing cascade in diabetic patients, a thermosensitive hydrogel incorporating stromal cell-derived factor-1 (SDF-1) and metformin, encapsulated in microspheres, was developed [46]. The initial release of SDF-1 accelerated the recruitment of bone marrow stem cells to the defect, and the delayed and subsequent release of metformin restored impaired osteogenesis by eliminating reactive oxygen species (ROS) [47]. In parallel efforts targeting diabetes, interleukin-4 was loaded into heparin-based microspheres to provide sustained immunomodulation and steer macrophage polarization toward pro-regenerative phenotypes [48].

Another important aspect of bio-inspired materials is the capacity to emulate the sophisticated characteristics and functional architectures found in nature. Electrically assisted 3D printing was employed to produce biomimetic Bouligand-type structures integrating modified multiwalled carbon nanotubes (MWCNTs). This approach allows precise orientation of the nanotubes, emulating the twisted plywood architecture found in natural fibers. This approach was used to build meniscus-resembling scaffolds with anisotropic mechanical properties [48]. In another example, the lotus root’s array of parallel, interpenetrating channels, which supports gas exchange and nutrient transport, has inspired ceramic scaffolds with lotus-root-mimetic microchannels that enhance intratissue revascularization for bone repair [49]. These nature-inspired materials open new possibilities for personalized, mechanically stable scaffolds for SGPR.

### Smart and Functional Materials

**Fig. 3:**
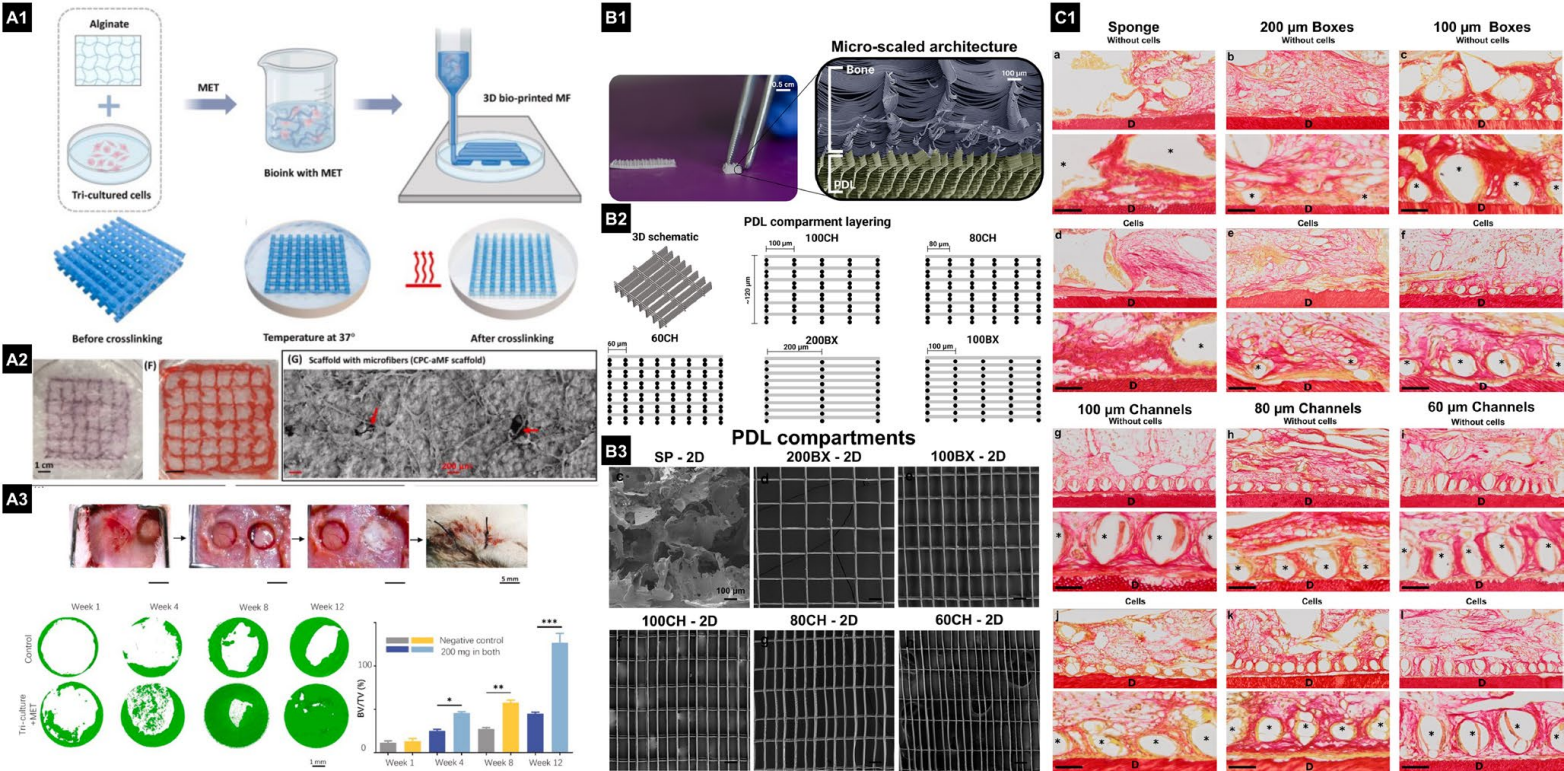
(**A1**) Schematic of the MEW setup used to fabricate poly(ε-caprolactone) (PCL) scaffolds with distinct fiber morphologies: random, aligned, and highly ordered 0°/90° crosshatch patterns with controlled strand spacing (250 μm and 500 μm). (**A2**) Representative SEM images of MEW scaffolds showing fiber architectures (random, aligned, crosshatch) with RAW 264.7 macrophages cultured on these scaffolds. Morphological differences are observed between spontaneously differentiated (round, M1-like) and LPS-stimulated (elongated, M2-like) macrophages, highlighting the influence of fiber orientation and spacing on cell phenotype. (**B1**) 3D defect visualization illustrating spatially zonal and tissue-specific scaffold considerations for periodontal regeneration. (**B2**) Masson’s trichrome-stained histological sections of periodontal defects treated with control (sham), collagen, aligned PDL scaffolds, coated fibrous scaffolds (F/CaP), and collagen-infused tissue-specific scaffolds, evaluated 6 weeks post-implantation. Yellow dashed lines mark scaffold placement and new bone formation areas; white arrowheads indicate the PDL region. Zones of new bone (NB), root surface (R), and soft tissue (St) are highlighted, demonstrating zonal-specific regeneration and scaffold integration. (**C1**) PCL/PEG/HA scaffolds with 200, 400, and 600 μm pores were characterized by photos and SEM. (**C2**) Micro-CT 3D reconstructions and sagittal images revealed incomplete mineralization in scaffold pores; new bone was indistinguishable from scaffold, so scaffold volume was measured pre-implantation to calculate bone volume fraction (BV/TV). BMD and trabecular thickness (Tb.Th) included both scaffold and bone. At 4 and 8 weeks, P600 scaffolds showed superior BV/TV, BMD, and Tb.Th, with bone defect areas highlighted. (**C3**) In vivo, macrophage responses (M1 CCR7⁺ green, M2 CD206⁺ red) at days 3, 7, and 28 showed fewer M1 macrophages around P600 scaffolds versus P200 and P400, while M2 numbers were similar at day 3. Both M1 and M2 increased over time, with the M2/M1 ratio highest in P600 scaffolds at days 7 and 28

Recent advances in materials science and biofabrication have yielded constructs that integrate with and adapt to the local microenvironment. This demand is driving the integration of CT-derived imaging to generate CAD models tailored to individual patients. Specifically for SGPR, image-based designs have been successfully translated from in vivo models [40] to a patient-specific scaffold [50]. Although PCL degradation caused failure at 14 months, the work established a workflow for creating personalized, functional scaffolds for periodontal regeneration. Additionally, CAD designs customized for MEW have been generated and digitally validated for accuracy and fidelity; however, reproducing complex internal morphology remains challenging (Fig. 3) [15]. Moreover, the advent of versatile 3D printing technologies like MEW has opened new possibilities for fabricating scaffolds that mimic native periodontal microenvironments and interfacial zones [6]. MEW scaffolds have been tested with various patterns and strand spacings to replicate bone and PDL compartments, optimizing the design for SGPR [15, 17, 51].

Beyond the functional outcomes enabled by printing technologies, the use of smart materials in regenerative strategies is an emerging area of research. Smart materials are biomaterials that respond to internal or external stimuli (e.g., pH, enzymatic activity, mechanical forces, or inflammatory cues) by adapting their physicochemical properties, degradation behavior, or bioactive release, thereby actively modulating the local regenerative microenvironment [52]. Through these adaptive responses to cellular and molecular signals, smart materials can provide enhanced guidance over tissue organization and repair. For example, combining polyisocyanide with ureido-pyrimidinone produces dynamic hydrogels that transduce mechanical inputs to support cell arrangement via mechanotransduction pathways [53].

These stimuli-responsive properties underpin the concept of 4D printing, a biofabrication strategy that extends conventional 3D printing by introducing time-dependent functionality. 4D printing refers to the fabrication of 3D-printed constructs using smart materials that undergo programmed, time-dependent changes in shape, properties, or functionality after printing, enabling dynamic adaptation to the biological environment [54–56] Given the structural and biological complexity of the periodontium, 4D printing of smart materials represents a promising approach to creating personalized scaffolds that respond to periodontal cues and support coordinated tissue regeneration.

## Cutting-Edge 3D Printing Technologies and Scaffold Design Principles

3D printing has transformed tissue engineering and regenerative medicine by enabling patient-specific scaffolds that replicate the architectural complexity of damaged tissues [57]. 3D printing for biomedical applications typically progresses in three phases: pre-printing, printing, and post-printing [58]. Before printing, the macro- and micro-scale features of the construct are defined. Medical imaging (e.g., computed tomography) provides datasets for computer-aided design (CAD), creating a precise digital model to guide fabrication [59, 60]. The printing phase involves three main steps: preparing bioink, selecting the printer, and executing the print. Success relies on optimizing the ink’s properties and design parameters. After printing, constructs are stabilized by crosslinking with ionic solutions or UV light to maintain integrity and functionality. Four main 3D bioprinting methods are used to create complex, anatomically accurate constructs: inkjet-based, extrusion-based, laser-assisted, and stereolithography [61]. All rely on coordinated toolpath control but differ in deposition methods: droplet ejection, continuous extrusion, laser transfer, or photopolymerization, impacting print fidelity, cell viability, and material compatibility.

### Extrusion-Based and Inkjet Printing

Advances in 3D printing enable the precise fabrication of complex scaffolds tailored to the requirements of biomaterials and tissues. Choosing the right technology depends on factors like printing resolution, shape fidelity, biomaterial type, and cell viability. Each technique has unique principles, capabilities, benefits, and limitations affecting tissue construct fabrication. Parameters such as temperature, speed, pressure, and nozzle characteristics impact shape fidelity and bioink printability. Additionally, bioink crosslinking type, concentration, and duration can be adjusted to optimize printing resolution and cell performance.

Printability, the ability of a bioink to form and retain the intended geometry during and after deposition, is a primary performance metric in bioprinting. It is governed by bioink viscosity, cell loading, scaffold stiffness, print temperature, and crosslinking kinetics [26]. Although higher viscosity improves filament formation and shape fidelity, it increases extrusion pressure and shear, which can risk cell damage. Shear-thinning bioinks are preferred as they reduce viscosity under stress, enabling smooth extrusion while maintaining structure.

Given its versatility and relevance to biofabrication, extrusion-based bioprinting has emerged as one of the most widely adopted techniques [62]. This method operates by continuously depositing bioink filaments in a layer-by-layer manner onto a substrate, guided by a robotic system through a nozzle [63]. Although generally more expensive than other approaches, extrusion-based bioprinters can process a wide range of bioink viscosities and concentrations and accommodate high cell densities [62, 63]. Illustrating this capability, Athirasala et al. (2018) extruded hybrid hydrogels comprising alginate, dentin-derived ECM, and stem cells from the apical papilla, producing cytocompatible scaffolds with inherent odontogenic potential [64]. Moreover, the literature has previously discussed the application of connective tissue matrix components and cell culture techniques in the biofabrication of oral soft tissues, including gingival constructs [65]. In this context, recent laboratory-based studies have demonstrated that polysaccharide-fibrinogen or collagen-based hydrogels, co-cultured with fibroblasts and/or endothelial cells and fabricated using extrusion-based 3D bioprinting, can successfully generate shape-controlled oral soft tissue (gingival) constructs [66, 67].

Despite its advantages, extrusion-based bioprinting poses some challenges. This process can expose encapsulated cells to mechanical stress and high pressure, potentially reducing cell viability to approximately 40–80% [68]. Moreover, achieving an optimal bioink viscosity is critical, not only to prevent nozzle clogging but also to ensure printing fidelity. Other limitations relate to the difficulty of maintaining the mechanical stability and integrity of large, free-form constructs [69]. Furthermore, this technique is characterized by relatively low resolution and slower printing speeds compared to other modalities. Nonetheless, extrusion-based bioprinting is well-suited for scalable production of complex constructs with varying properties, such as stiffness, porosity, and bioactivity [70, 71].

Conversely, inkjet printing [72], presents a different set of strengths and limitations compared to other methods and has been applied more selectively in regenerative dentistry research (Table 1). In summary, this approach supports the production of complex, multi-material constructs with high spatial precision. Some advantages include cost-effectiveness, rapid processing, superior resolution, and high cell viability (exceeding 85%) [68, 73]. Furthermore, it enables the accurate placement of biological agents to create tailored 3D tissue constructs. However, its efficiency decreases when printing highly viscous inks, which limits its suitability for certain materials [63].

**Table 1:** Comparison of inkjet- and extrusion-based printing techniques. Abbreviations: ~: approximately, mPa.s: millipascal-second, µm: micrometer

Features	3D-Priting techniques	
	Inkjet printing	Extrusion-based printing
*Development*	1998–2002	2002
*Mechanisms*	Thermal, piezoelectric, electrostatic, and electrohydrodynamic	Pneumatic, piston, and screw
*Printer cost*	Low	Medium
*Resolution*	~ 30 μm	100 μm
*Fabrication Speed*	Low	medium
*Support structure required*	Yes	No
*Advantages*	Fabrication of high-cell-density scaffolds; compatible with inks across a wide viscosity range (30 to 6 × 10^7^ mPa.s); suitable for in situ printing; and large-scale production.	Low cost; high compatibility; rapid fabrication; superior resolution; and easy bioink replacement, enabling the creation of fine structural details.
*Disadvantages*	Requires longer crosslinking time; cell damage; low resolution (5–100 μm); and slow printing speed.	Nozzle clogging using high cell densities; low cell-density; inability of the printhead to maintain continuous flow; limited resolution; low mechanical properties; and restricted compatibility with high-viscosity materials (3.5–12 mPa.s).
*Main dental applications*	Dentin-pulp complex [76–79]; cementum [21, 22]; alveolar bone, gingiva, and/or PDL regeneration [22, 23, 39, 73]; tooth and root-like structures in vivo [75, 80].	Dental pulp-like tissue [81], PDL [82] gingival grafts [66, 67], and alveolar bone regeneration [16, 83–85].

Inkjet-based printing technologies are generally classified into continuous inkjet (CIJ) and drop-on-demand (DOD) systems [73]. CIJ generates a steady stream of ink droplets using high-frequency pulses; these droplets are charged and deflected to control deposition. CIJ is mainly used for large-scale production where high printing precision is not required [72]. In contrast, the latter operates without droplet recovery, offering a simpler design and precise control over droplets. DOD has become the predominant inkjet modality, using thermal, piezoelectric, electrostatic, or electrohydrodynamic actuation to generate fine bioink droplets [73]. Compared with extrusion printing, inkjet approaches have been less explored for oral tissue regeneration. Laboratory-based studies on extrusion-driven 3D printing have primarily focused on regenerating the dentin-pulp complex in vitro, reconstructing the periodontal complex (including gingiva, cementum, and alveolar bone) [70, 74], and demonstrating the potential to regenerate entire tooth-like structures in vivo [75]. Across these studies, bioinks typically include stem cells sourced from the target tissue and growth factors to enhance differentiation and mineralization.

### Bioprinting of Functionalized Constructs

3D bioprinting enables precise, uniform placement of cells, ECM components, and bioactive molecules within customizable constructs [86]. It creates 3D living constructs that mimic physiological conditions, maintain long-term cell viability, and replicate native tissue functions. During the bioprinting process, several factors can influence cell viability in the fabricated scaffolds, including the rheological properties of the bioink, its biocompatibility, biodegradability, and porosity [3]. Increased research efforts have focused on the fabrication of large, functional tissue constructs featuring biomimetic vascular networks and micro- and nanoscale architectures. These efforts aim to replicate the complexity of native tissues, such as the pulp-dentin complex [78, 79, 81] and the periodontium [35, 87, 88], which exhibit intricate structural and biological features crucial for their regeneration.

Nonetheless, selecting biomaterials with suitable mechanical stability, degradation profiles, and compatibility with specific 3D printing techniques remains difficult [89]. Therefore, designing functional physiological structures that integrate and address the natural transitions between different tissues remains a major focus of research.

Polymers are the predominant class of materials used in bioinks and scaffold creation. Among these, hydrogels, with their hydrophilic polymer chains linked by crosslinks, closely resemble the native ECM. Their porous structure allows the diffusion of small molecules, making them highly useful as structural supports in tissue engineering. Laboratory studies have shown that polymer-based bioinks (e.g., collagen, gelatin methacrylate, polycaprolactone, alginate, and fibrinogen), when combined with stem cells or bioactive factors, can promote spatially controlled cell differentiation [22, 39] and the formation of mineralized [77, 79], vascularized dental pulp-like tissue [81], as well as structures similar to PDL and alveolar bone [22, 40]. Among the most common constructs, polymeric bioinks with human dental pulp stem cells (hDPSCs) have been extensively studied for dental pulp regeneration [78, 81, 90]. In contrast, bioinks with human PDLSCs are often used for periodontal regeneration (Fig. 3) [15, 22]. Additionally, acellular matrices enriched with bioactive agents have also shown promising results in boosting the differentiation of local stem cells, thereby aiding the reconstruction of functional tooth-supporting tissues [75, 88, 91]. Regarding soft tissues biofabrication, although significant progress has been made, challenges such as reproducing the complex microarchitecture of native tissues, maintaining long-term viability and integration of printed constructs in vivo, and scaling up for clinical use remain [92]. For instance, future studies should investigate the incorporation of microvascular networks and multimaterial, multiphasic bioprinting strategies embedding bioactive factors, followed by in vivo implantation, to demonstrate effective support for immune modulation and infection control in the development of vascularized mucogingival grafts.

### In Situ 3D Printing

The most common method for creating implantable tissues involves growing cells on 3D scaffolds in the lab. However, this has limitations, including reduced long-term cell survival, scaffold degradation before implantation, and a higher risk of contamination [93, 94]. Moreover, the final shape and size of the engineered construct may not perfectly match the defect site, which can lengthen surgery time and increase the risk of gaps or damage to the scaffold, leading to more invasive procedures [94]. In this context, the idea of in situ 3D bioprinting was first introduced in 2007, with the proposal to use robotic inkjet bioprinting to directly deposit bioink into calvarial defects [95, 96], marking a major step forward in tissue engineering and regenerative medicine. This method introduces minimally invasive procedures by allowing precise placement of cells, biomaterials, and bioactive molecules directly at the target site with a bioprinter [96]. As a result, it provides highly personalized, patient-specific treatment, enables rapid wound coverage, and has great potential to create structures with specific shapes tailored to the anatomical needs of the area.

The bioprinters used for in situ 3D printing are generally divided into handheld devices (portable and custom tools that can be operated manually, providing greater flexibility) and robotic systems. The handheld bioprinting tool offers several benefits over robotic systems, including the ability to handle time-sensitive wound conditions, deposit bioinks into defects without support, a smaller size, and much lower costs. However, this method is limited by its low resolution and lack of precise spatial control [97]. Unlike traditional 3D printing, robotic-assisted devices use a portable three-axis system to guide the bioprinting head, with slicer software determining the printing path, which reduces unintended hand movements during operation [93, 97]. To ensure accurate deposition of cell-laden bioinks, this technology must combine precise XYZ-axis positioning, automated biomaterial dispensers, and easy-to-use software [93]. Although the robotic method still requires expertise for calibration and print planning.

While in situ bioprinting has progressed in regenerating skin, cartilage, muscle, and bone, it remains largely underexplored in regenerative dentistry. Using a handheld bioprinter, Duarte Campos et al. [81] injected a collagen-based bioink loaded with cells for dental pulp tissue regeneration inside root canals in vitro [81]. Immunofluorescence analysis showed that the injected scaffolds promoted successful vascularization without bioink shrinkage. Additional validation in animal models is needed, as demonstrated by the in-situ regeneration experiments conducted by Qian et al. [90], in which minipigs’ incisors developed highly vascularized pulp-like tissue and uniformly arranged odontoblast-like cells after treatment with gelatin methacryloyl (GelMA) microspheres loaded with hDPSCs [90]. Proper training in bioprinting operation is crucial to maintain tissue integrity, alongside selecting biocompatible models and bioinks with suitable mechanical properties.

### Hierarchical and Anisotropic Structures

Replicating the hierarchical, anisotropic structure of dental tissues is a major challenge in functional regeneration. These tissues have multiscale organization within a specialized ECM that influences cell behavior in 3D [98]. Anisotropic, multiphasic periodontal scaffolds are engineered to mimic the complex architecture and functional zones of the periodontium; alveolar bone, cementum, and PDL. They feature directional properties like aligned fibers or stiffness gradients to guide cell orientation and tissue regeneration, and consist of distinct material phases that replicate the native tissue heterogeneity [43, 44, 99]. Conventional clinical methods often overlook this complexity, limiting effective restoration. Even with 3D bioprinting, accurately reproducing the native hierarchy and biomechanical properties with sufficient spatial precision is difficult [100]. This challenge is particularly significant for the pulp-dentin complex, PDL, and alveolar bone, where orientation-dependent mechanics and biology operate across macro- to nanoscales [101].

Hierarchically graded tissues feature a spatially controlled distribution of different cell types, ECM components, and functional elements, arranged in an ordered manner across macro- to microscale gradients that mirror the complexity of physiological tissues. In these systems, anisotropy is not just a structural feature but also serves an instructive role, affecting cell behavior, tissue organization, and regenerative outcomes. Consequently, scaffold-guided cell alignment has become a critical design approach, especially in neural and musculoskeletal tissue engineering, where directional cues are vital for promoting functional recovery [101]. Among extrusion-based methods, coaxial bioprinting has been extensively explored in vascular tissue engineering, enabling the creation of constructs with both internal and external hierarchical structures [102, 103]. Additionally, combining extrusion-based printing with microfluidic systems allows precise control over the structural and compositional features of engineered tissues [104, 105].

Specialized bioinks and advanced bioprinting are key to high-precision, factor-integrated cell patterning that recreates anisotropic, complex tissues. By adjusting parameters such as nozzle diameter, deposition patterns, and printing speed, it is possible to engineer constructs that mimic natural gradients and directional properties, thereby encouraging cell adhesion, proliferation, and migration. Importantly, multiphasic scaffolds with graded mechanics more accurately match the mechanical heterogeneity of host tissues, enhancing integration and function [105]. Layer-specific design enables targeting of PDL, alveolar bone, cementum, and gingiva within a single construct. In parallel, anisotropic hydrogels with programmable polymer alignment offer superior mechanical performance compared to conventional hydrogels and replicate the aligned microenvironments of native ECM [106, 107]. Advances in phase separation and multimaterial strategies now produce interconnected, porous, and anisotropic architectures that improve oxygen and nutrient transport, as well as guide directional cell migration and tissue ingrowth [108, 109]. Overall, these tunable, high-porosity systems hold strong promise for regenerative medicine, drug screening, and personalized therapies.

Shao et al. (2024) identify three main challenges for 3D bioprinting of hierarchically anisotropic constructs: aligning hydrogel molecular chains to create anisotropy, increasing scaffold porosity, and improving the printability of low-viscosity hydrogels while preserving mechanical stability [110]. Addressing these challenges is crucial for clinical use of bioinspired anisotropic scaffolds.

## Biological Performance and Regenerative Outcomes

Periodontal regeneration requires coordinated repair of cementum, PDL, alveolar bone, and gingiva-tissues with distinct biology. As discussed, 3D printing with image-based CAD and bioactive or stimuli-responsive materials enables the fabrication of multiphasic scaffolds that mimic native structure, guide cell growth, and deliver therapeutics. Table 2 summarizes the principal materials employed in 3D printing for periodontal regeneration and highlights their respective applications across different periodontal tissues. This section reviews recent advances in 3D-printed scaffolds for functional periodontium reconstruction and explores related fields to enhance translation.

**Table 2:** Summary of materials and their respective applications in 3D printing for periodontal regeneration

Material system	3D printing strategy	Key design / functional strategy	Bioactive components (examples)	Main biological outcome
Polycaprolactone (PCL)	Melt electrowriting (MEW)	Controlled fiber alignment and strand spacing (≈ 300–500 μm)	—	Guided periodontal ligament (PDL) fiber orientation, osteogenesis, reduced inflammatory response
PCL-based multiphasic scaffolds	Melt electrowriting (MEW)	Spatially graded architectures mimicking bone–PDL compartments	Calcium phosphate coatings	Simultaneous mineralized tissue formation and functional PDL attachment
Polymers / hydroxyapatite composites	Extrusion-based printing	Porosity control and surface exposure of ceramic phase; optimization of pore size (~ 600 μm)	Ca^2+^/PO_4_^3−^ ions	Enhanced osteogenic differentiation, osteoconduction, modulation of macrophage response, vascularization and bone formation
GelMA / alginate hydrogels	Extrusion bioprinting	Cell-friendly hydrogel matrix with reinforced mechanical stability	β-TCP, bioactive glass	Improved printability, osteogenic differentiation, and mineral deposition
Extracellular matrix (ECM)-based hydrogels	Extrusion bioprinting	Biomimetic extracellular matrix environment	RGD (Arg-Gly-Asp) motifs and ECM-derived cues	Improved cell infiltration, proliferation, vascularization, and osteogenic differentiation
Amorphous magnesium phosphate–functionalized scaffolds	Melt electrowriting (MEW), extrusion bioprinting	Integration of ion-releasing phases within fibers and/or fillaments	Mg^2+^/PO_4_^3−^ ions	Improved osteogenic differentiation, mineral deposition, and bone formation
Calcium phosphate ceramic inks	Extrusion-based printing	Patient-specific based scaffold design	Ca^2+^/PO_4_^3−^ ions, Sr^2+^ (in selected systems)	Precision fit, cytocompatibility, improved cell infiltration, proliferation, and enhanced bone regeneration
Polymeric scaffolds with drug delivery capability	Extrusion-based printing	Incorporation of therapeutic agents within scaffold matrix	Antibiotics, statins, growth factors (e.g., BMP-2)	Antimicrobial activity, immunomodulatory effects (M1 and M2 polarization), and enhanced regenerative outcomes

### Cellular Interactions and Tissue Integration

Guided periodontal regeneration is successful when scaffolds replicate the structural and biochemical environment of the periodontium to promote cell adhesion, proliferation, and differentiation. Material composition, surface topology, and porosity influence cell behavior and healing. Integrating microscale architectural cues with ECM-inspired biochemical functionalization encourages PDL fiber alignment, osteogenic differentiation, and vascularization, which are essential for effective regeneration. The growth of PDLSCs and the increased expression of osteogenic markers such as ALPL, OCN, and RUNX2 were stimulated by collagen-based bioinks (10 mg/mL), providing an ECM-like environment that supports PDLSC migration and alignment along root dentin surfaces in vivo [42]. Structural guidance paired with biochemical cues has been shown to regenerate both ligament and bone tissues [111]. Alginate matrices functionalized with arginine-glycine-aspartic acid (RGD) domains, which mimic ECM binding motifs, were shown to enhance the viability, migration, and osteogenic differentiation of bone marrow stem cells cultured on rigid 3D-printed hydroxyapatite scaffolds with osteoid-like stiffness [112].

Other studies have examined how topographical features influence cell behavior and immune responses. Alkaline hydrolysis (NaOH treatment) of PCL/hydroxyapatite 3D-printed scaffolds reduced foreign body reactions and promoted M2 macrophage polarization in vivo, resulting in enhanced bone formation [113]. This demonstrates that simple surface modifications can enhance biological outcomes. Another approach focused on pore size and fiber/filaments orientation. Aligned MEW PCL fibers with large strand spacing (500 μm) increased the gene expression of RUNX2 and IL10 while decreasing pro-inflammatory markers in vitro [15]. Similarly, polycaprolactone/polyethylene glycol/hydroxyapatite (PCL/PEG/HA) scaffolds printed by extrusion with 600 μm pores reduced inflammation, increased M2 macrophage polarization, and supported vascularization and bone growth (Fig. 3) [113]. Moreover, hydroxyapatite and polylactic acid (HA-PLA) composite scaffolds printed by extrusion with lower infill density (i.e., higher porosity) enhanced human osteoblast-like cell proliferation and protein synthesis, with increased protein concentration of cytokines IL-6 and IL-8 [114]. These findings emphasize that scaffold properties impact cell proliferation and modulate the immune response, key factors in bone healing.

**Fig. 4:**
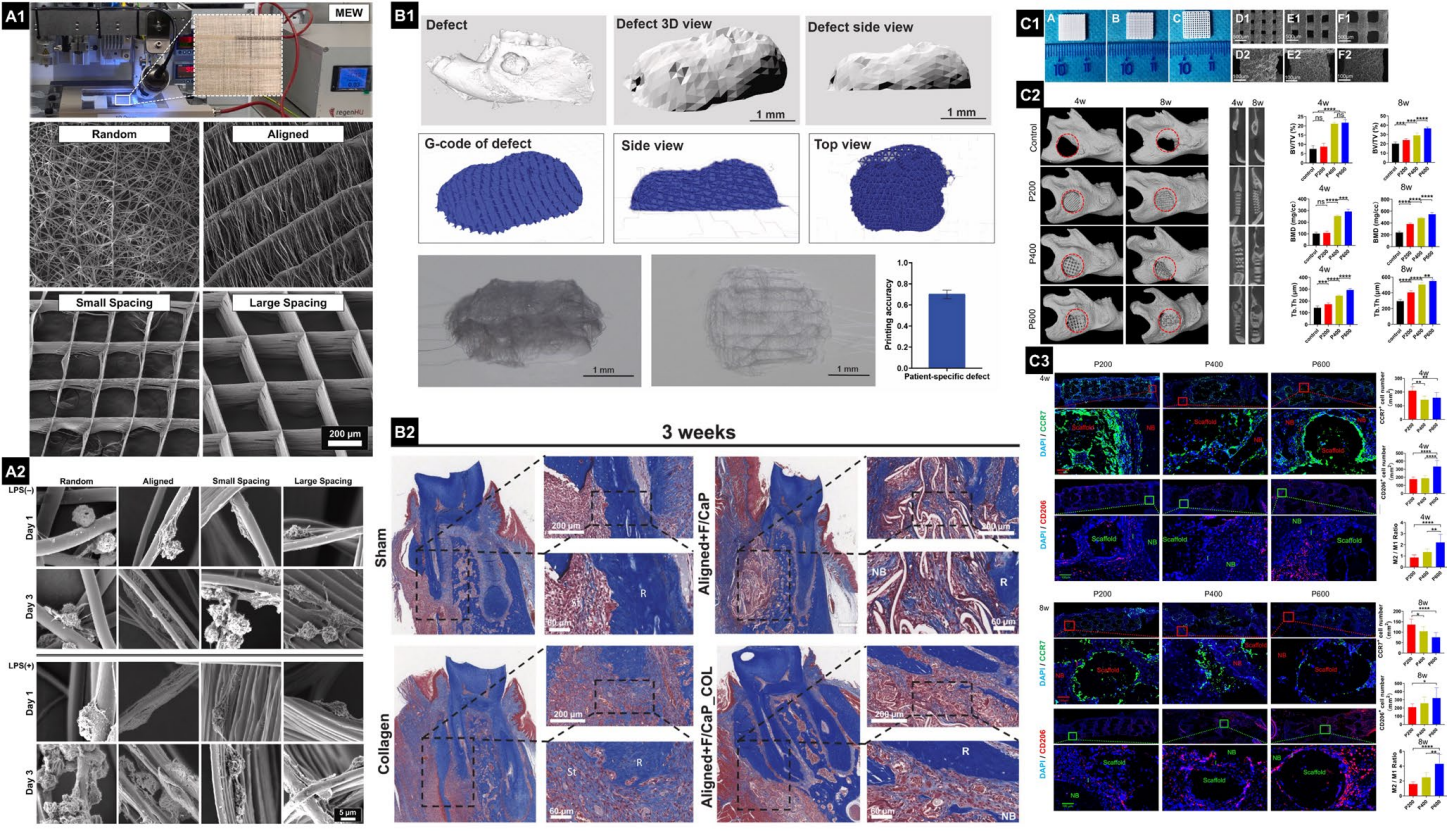
(**A1**) Fabrication of a 3D bioprinted aligned microfiber (aMF) system encapsulating a human periodontal ligament stem cell (hPDLSC)-based tri-culture within a calcium phosphate cement (CPC) scaffold for enhanced bone regeneration. The illustration outlines the design and composition of the bioink, along with a computer-aided 3D model of the construct. The bioprinting process, based on the FRESH technique, enables precise deposition of the aMF carrier system. (**A2**) Macroscopic images demonstrate the gross morphology of the printed constructs (scale bar = 1 cm), while scanning electron microscopy (SEM) reveals the presence of interconnected macropores that facilitate tri-culture cell infiltration and outgrowth. (**A3**) In vivo osteogenic evaluation of 3D-bioprinted aMF-CPC scaffolds. A schematic outlines the rat bilateral cranial defect model and surgical workflow, with groups receiving either no implant (control) or scaffolds encapsulating tri-culture cells plus metformin (scale bar, 1 cm). Micro-CT reconstructions depict the defect region (scale bar, 1 cm), and bone regeneration is quantified as bone volume to total volume (BV/TV) within the defect at 1, 4, 8, and 12 weeks. (**B1**) Benchtop image of the MEW scaffold with an enlarged inset showing the biphasic scaffold’s internal micro-architecture-bone compartment (blue) and PDL compartment (green). (**B2**) 3D schematic of the PDL compartment with cross-sectional diagrams of all configurations, including 100 μm channels (100CH), 80 μm channels (80CH), 60 μm channels (60CH), 200 μm boxes (200BX), and 100 μm boxes (100BX). (**B3**) SEM images of fabricated PDL compartments: salt-leached sponge (SP-2D), 200 μm boxes (200BX-2D), 100 μm boxes (100BX-2D), 100 μm channels (100CH-2D), 80 μm channels (80CH-2D), 60 μm channels (60CH-2D). (**C1**) Histology of dentine slice/scaffold implants 8 weeks post-implantation in a rodent model. Collagen fibers (stained with Picrosirius Red) densely populated the PDL compartments in all groups. Fiber alignment mirrored the nuclei orientation and depended on the presence of fiber-guiding features. Control groups (SP, SP + C, 200BX, 200BX + C) showed mainly parallel collagen fibers attached to dentine, while fiber-guiding groups with ≤ 100 μm spacing consistently exhibited oblique and perpendicular collagen alignment to the dentine

Multicompartment scaffolds have also been developed to target different tissue types simultaneously. MEW PCL scaffolds supported long-term 3D coculture of osteoblasts and PDL fibroblasts, resulting in a continuous mineralized gradient and PDL-like insertions [99]. This was further expanded with a biphasic MEW scaffold featuring a pore-size gradient in the bone compartment and microchannels (≤ 100 μm) to guide fiber orientation in the ligament region [51]. These features led to over 60% of collagen fibers aligning with the channel direction in vitro and promoted the functional attachment of oblique fibers, as well as periostin expression in vivo (Fig. 4) [51]. These studies demonstrate that scaffold architecture influences cell behavior, modulates inflammation, and facilitates bone-ligament regeneration. Well-designed 3D-printed scaffolds actively shape the healing environment, improving outcomes in periodontal regeneration.

### Delivery of Therapeutics and Microenvironment Modulation

Besides architectural features, the orchestration of periodontal regeneration critically depends on bioactive cues that modulate cellular behavior, immune responses, and ECM remodeling. In this context, the controlled delivery of therapeutic agents from 3D-printed scaffolds emerges as a pivotal strategy to modulate the complex microenvironment of the periodontium. Many studies have focused on harnessing the osteoinductive and angiogenic potential of bioactive ions released from biomaterials. Bioactive glasses and calcium phosphate-based materials serve not only as structural scaffolds but also as reservoirs of ions such as Si^4+^, Ca^2+^, Mg^2+^, and Sr^2+^, which exert a profound effect on cell behavior and tissue mineralization. Mesoporous bioactive glass nanoparticles, incorporated into GelMA 3D-printed hydrogels, greatly improved scaffold shape fidelity and surface roughness, leading to better adhesion, spreading, and osteogenic/cementogenic differentiation of PDL cells [115]. Similarly, MEW PCL scaffolds functionalized with 5% 58 S-bioactive glass significantly enhanced the viability, spreading, and mineral matrix deposition of aBMSCs compared to pristine PCL scaffolds, while also improving angiogenesis in vivo [18]. Consistent with these results, β-TCP has been shown to improve the printability, mechanical stability, bioactivity, and structural integrity of GelMA/alginate hydrogel-based bioinks [35]. This formulation improved shear-thinning behavior, mechanical strength, ionic crosslinking, and bioactivity, supporting the adhesion and osteogenic differentiation of aBMSCs and maintaining biocompatibility [35]. In vivo, constructs loaded with β-TCP showed no signs of inflammation after three weeks, and calcium carbonate deposits confirmed β-TCP’s potential to promote mesenchymal stem cell differentiation into a mineralizing phenotype (Fig. 2A) [35].

Expanding the range of therapeutic ions incorporated into calcium phosphate systems, the use of Pluronic F127 and β-TCP-based ceramic inks containing 5% strontium carbonate (SrCO_3_) produced Sr-BCP scaffolds with enhanced mineralized nodule formation and improved mechanical strength, whereas β-TCP controls maintained higher viability of aBMSCs [116]. This suggests that although Sr-enrichment enhances mechanical and mineralization outcomes, preserving the intrinsic cytocompatibility of β-TCP may be advantageous in clinical scenarios where maintaining high cell viability is crucial, such as during the early stages of healing. This ink formulation strategy offers a viable pathway to tailor CaP scaffold performance and can be extended to other therapeutic ions [116]. In the same direction, the combination of alginate/β-TCP and hydroxyapatite/α-TCP, explored through a clinically feasible workflow integrating cone-beam computed tomography (CBCT)-based design and OsteoInk™ 3D printing, yielded precision-fit scaffolds for alveolar bone reconstruction. Both compositions were cytocompatible, and the hydroxyapatite/α-TCP composition showed superior viability of aBMSCs [12]. Also, the strategic incorporation of magnesium and strontium ions within calcium silicate matrices finely modulates the scaffold’s ionic dissolution kinetics and degradation profile, achieving an optimized balance between bioactivity and structural integrity that enhances osteoinductivity and angiogenic capacity [117]. These ions, known for their osteogenic signaling properties, contribute to enhanced osteogenic activity, ECM mineralization, and endothelial network formation, underlining the multifaceted roles of ionic cues in periodontal regeneration.

AMP are known for their osteoconductive and bioactive properties and have been used in hydrogel and composite scaffolds to enhance mineralized tissue regeneration. Printed hydrogels composed of ECM and AMP promoted an elongated morphology and enhanced osteogenic differentiation of dental pulp stem cells (DPSCs), even in the absence of osteogenic supplements. In vivo experiments confirm increased bone density and volume over 4 to 8 weeks [16, 116]. Meanwhile, the crystalline form of magnesium phosphate was incorporated into MEW PCL fibers, which supported the osteoblastic differentiation of human mesenchymal stem cells in vitro and enhanced in vivo bone formation in a rat mandibular fenestration model [17]. Notably, the fiber alignment within these scaffolds also facilitated PDL regeneration, demonstrating that ionic bioactivity and scaffold architecture synergistically promote regeneration. In load-bearing applications, AMP has also been incorporated into polyetheretherketone (PEEK) composites, creating a material with controlled hydrolytic degradation that achieves osseointegration similar to that of the gold-standard titanium implants used in clinics [118]. Collectively, these studies demonstrate the potential of magnesium phosphate bioceramics as a versatile, ion-releasing component that promotes osteogenic differentiation and enables controlled scaffold degradation and supports the regeneration.

Additional studies with magnesium-doped calcium silicate scaffolds showed favorable degradation profiles and significantly higher osteogenic potential compared to standard tricalcium phosphate and other bioceramics, highlighting the importance of controlled ion release kinetics for long-term scaffold performance [119]. Likewise, calcium phosphate cement scaffolds combined with calcium phosphate ion-releasing hydrogels have been shown to enhance bone, vascular, and nerve regeneration in cranial defect models, further highlighting the combined roles of bioactive ions in complex tissue regeneration (Fig. 4) [120]. These findings collectively emphasize that modifying ionic composition and release from biomaterials can precisely regulate cellular differentiation, matrix mineralization, and angiogenesis, all of which are essential for effective periodontal and alveolar bone regeneration.

Complementing ion delivery, the controlled release of growth factors is also proposed to boost regeneration by directly stimulating cellular proliferation, differentiation, and neovascularization. Miao et al. (2023) developed GelMA/sodium alginate/bioactive glass microsphere 3D-printed hydrogels loaded with mesenchymal stem cells and dual growth factors BMP-2 and PDGF. Their system showed improved osteogenic differentiation, apatite formation, and mechanical strength in vitro, which led to substantial regeneration of gingival tissue, PDL, and alveolar bone in a canine periodontal defect model [70]. In a similar approach, PCL/β-TCP scaffolds functionalized with BMP-2 or supplemented with autogenous bone particles promoted increased bone volume in vivo in a canine model [121]. Another strategy involved PLA printed scaffolds surface-functionalized with polyethyleneimine (PEI) and VEGF-encoding plasmid DNA nanoplexes (pVEGF) [122]. This platform exhibited high encapsulation efficiency and sustained release, upregulating osteocalcin and VEGF expression, promoting tube formation in vitro, and enhancing new bone formation and vascularization in rat calvaria defects [122].

Immunomodulation through scaffold design and drug delivery has been identified as a vital aspect of periodontal regeneration, given the crucial role of inflammation resolution and macrophage polarization in tissue repair and regeneration. The shift of macrophages from a pro-inflammatory M1 phenotype to a pro-regenerative M2 phenotype creates a favorable environment for regeneration by modulating cytokine profiles and recruiting progenitor cells. Multifunctional 3D-printed scaffolds combining PCL, chitosan, L-arginine, and β-TCP effectively decreased pro-inflammatory cytokines, such as IL-6 and TNF-α, while increasing osteogenic growth factors, BMP-2 and TGF-β, in macrophages [123]. This synergistic modulation facilitated a phenotypic shift from M1 to M2 macrophages while enhancing osteogenic mineralization, underscoring the ability of composite biomaterials to actively coordinate the immune environment and mineralized tissue regeneration [123]. Similarly, a 3D bioprinted hydrogel microfiber system encapsulating human PDLSCs in a tri-culture model within a calcium phosphate cement scaffold featuring a two-stage metformin release was shown to support complex interactions among hPDLSCs, endothelial cells, and pericytes, creating a microenvironment conducive to the regeneration of bone, vasculature, and nerve tissues (Fig. 4) [120].

Besides promoting regeneration, tackling microbial challenges remains a vital concern for periodontal biomaterials. A GelMA-printed membrane loaded with chitin whiskers and lipid nanoparticles co-delivering simvastatin and grape seed extract not only promoted osteogenesis in human mesenchymal stem cells but also prevented bacterial contamination by *Streptococcus mutans* and *Porphyromonas gingivalis in vitro* [124]. Importantly, transplanting this scaffold in a rat periodontal disease model significantly enhanced alveolar bone regeneration while reducing acute inflammatory responses, demonstrating the potential of combined osteoimmunomodulatory and antimicrobial strategies for effective periodontal regeneration. Attenuation of *P. gingivalis* growth was achieved by MEW PCL scaffolds coated with nanostructured fluorinated calcium phosphate, with in vivo implantation confirming biocompatibility and periodontal regeneration in a rat mandibular fenestration defect model [14]. Similarly, incorporating tetracycline hydrochloride into 3D-printed PCL scaffolds effectively inhibited the growth of Staphylococcus aureus while promoting mineralization [125].

## Challenges and Future Perspectives

Despite major advances, several barriers still restrict the routine clinical application of 3D-printed scaffolds for periodontal regeneration. The primary challenge is vascularization: approaches such as prevascularization, angiogenic factor delivery, and microchannel design show promise; however, creating stable, perfusable networks remains challenging [126]. Reproducing tissue heterogeneity and graded interfaces across cementum-PDL-bone is also technically complex, and many constructs still face challenges in coordinating regeneration across different tissue interfaces [127]. Integration of antimicrobial functionality is critical, as scaffolds must prevent bacterial colonization while supporting tissue repair [1, 2]. Strategies include embedding antibiotics, metal ions, or antimicrobial peptides, designing biofilm-resistant surfaces, and developing stimuli-responsive release systems. Ensuring long-term efficacy without compromising cell compatibility or scaffold performance remains challenging. Since chronic inflammation is inherent to periodontitis, scaffolds must actively influence immunity; immuno-instructive matrices and anti-inflammatory/cytokine delivery are promising but require rigorous validation [49]. Further hurdles in translation also exist: the complexity of cell-laden, bioactive, and biodegradable systems complicates manufacturing control (including consistency and sterility) and alignment with changing regulatory standards [122]. Clinical trials demonstrating safety and effectiveness are still limited [123, 128].

Machine learning (ML) and artificial intelligence (AI) offer opportunities beyond personalization, including optimization of printing parameters, scaffold fit, material deposition, and prediction of print fidelity and mechanical performance [66, 129, 130]. AI-assisted pipelines can integrate defect morphology, materials, and biological outcomes, reduce trial-and-error, and improve scaffold design. Future advances like machine-learning-guided design, hybrid bioprinting, and prevascularized immunomodulatory constructs will enhance capabilities. Despite rapid progress, clinical translation of 3D-printed periodontal scaffolds is constrained by three interdependent barriers. Manufacturing and quality challenges include achieving reproducible microscale architectures across prints, validating patient-specific digital workflows, defining critical quality attributes (e.g., pore fidelity, mechanical performance, degradation behavior), and ensuring sterilization compatibility and shelf-life without compromising bioactivity. Regulatory and safety problems become more complex as scaffolds incorporate bioactive agents, living cells, or gene/drug delivery, raising questions of product classification (device vs. combination product), required preclinical safety packages, and control of risks related to degradation byproducts, residual solvents/monomers, endotoxin/bioburden, and extractables/leachables. Clinical implementation barriers include the heterogeneity of periodontal defects and patient risk factors, the need to function in a bacteria-rich oral environment under occlusal loading, surgical handling and fixation/space maintenance, and the challenge of selecting meaningful long-term clinical endpoints that reflect true functional attachment rather than short-term defect fill.

## Conclusion

3D printing is reshaping periodontal regeneration by enabling patient-specific, multiphasic scaffolds that emulate native architecture and mechanics while supporting cell adhesion and differentiation, and tissue regeneration. Combining precise fabrication with bioactive and stimuli-responsive materials enables the spatial control of biological cues, thereby enhancing functional outcomes. Major challenges include achieving stable vascularization, immune modulation, scalable manufacturing with strict quality control, and clear regulatory pathways. Promising directions involve prevascularized and immuno-instructive, multi-compartmental designs; hybrid/4D bioprinting; and data-driven (e.g., machine-learning-guided) optimization. Ongoing collaboration among materials scientists, bioengineers, clinicians, and regulators, with thorough clinical validation, is crucial for developing safe, effective, personalized periodontal treatments.

## Data Availability

No datasets were generated or analysed during the current study.
